# Low-Carbon Valorization of Construction Spoil into High-Value Recycled Aggregates for Geopolymer Concrete

**DOI:** 10.3390/ma19050922

**Published:** 2026-02-27

**Authors:** Lei Zhang, Kai Wang, Yuting Gao, Xiaoxiong Zha, Yu Lei

**Affiliations:** 1 Shenzhen Construction Engineering Group Co., Ltd., Shenzhen 518055, China; zl_zrh2018@163.com (L.Z.); gaoytcn@163.com (Y.G.); 2 School of Intelligent Civil and Ocean Engineering, Harbin Institute of Technology, Shenzhen 518055, China; 3 Guangdong Provincial Key Laboratory of Intelligent and Resilient Structures for Civil Engineering, Shenzhen 518055, China; 4 China Jiangxi International Economic and Technical Cooperation Co., Ltd., Dar es Salaam P.O. Box 70299, Tanzania

**Keywords:** construction spoil (CS), geopolymer concrete, recycled aggregate, compressive strength, low-carbon construction

## Abstract

This study investigates the production of recycled aggregates (RAs) derived from construction spoil (CS) and their influence on the mechanical properties of geopolymer concrete. Two manufacturing routes, disc pelletization and crushing granulation, were employed to produce CS-based RAs. The resulting RAs were characterized in terms of particle size distribution and geopolymer compressive strength development. Geopolymer concretes incorporating disc-pelletized and crushed aggregates achieved 7-day compressive strengths of 31.0–32.5 MPa and 37.9–38.4 MPa, 21-day compressive strengths of 31.6–36.5 MPa and 40.8–41.5 MPa, 28-day compressive strengths of 36.9–37.1 MPa and 42.3–43.5 MPa, respectively. These results confirm the technical feasibility of using CS as a high-value RA resource in structural geopolymer concrete. At the same time, the approach offers environmental and economic benefits by reducing the reliance on conventional natural aggregates and lowering the associated carbon footprint. Compared with disc-pelletized RAs, crushed RAs exhibit superior performance in improving concrete compressive strength, which is attributed to their angular morphology and higher apparent density that enhance the overall structural integrity of the concrete matrix. In contrast, disc-pelletized RAs display higher porosity and smoother surfaces, which tend to induce stress concentration and thus reduce the mechanical performance of geopolymer concrete. Overall, the findings provide practical guidance for the valorization of construction spoil through RAs production. They demonstrate that crushed CS-derived RAs can effectively replace natural aggregates in structural concrete, thereby mitigating the impacts of aggregate mining and contributing to circular economy and low-carbon construction objectives.

## 1. Introduction

The rapid urbanization worldwide has spurred unprecedented growth in the construction industry, yet it has also led to severe environmental and resource challenges. On one hand, the extensive use of cement-based concrete contributes significantly to global CO_2_ emissions [1], contradicting the principles of low-carbon development. On the other hand, the heavy reliance on conventional natural aggregates in civil engineering exacerbates ecological degradation due to excessive quarrying, leading to landscape destruction and biodiversity loss. Moreover, large-scale excavation activities—such as foundation pit construction and subway tunneling—generate vast amounts of construction spoil (CS, e.g., excavated soil, demolition waste). Improper disposal of such waste not only occupies valuable land resources but also causes air and groundwater pollution, along with potential geohazards like mudslides and slope failures. Currently, CS recycling remain largely inadequate, falling into two problematic categories: low-value applications (such as backfilling or land reclamation) or energy-intensive processing (like sintering at high temperatures). This dichotomy presents a critical challenge—how to transform CS into high-performance construction materials while maintaining energy efficiency and environmental benefits.

Integrating CS recycling with recycled aggregate (RA) production presents a promising solution to these challenges. Recent advances in material science and waste valorization techniques have demonstrated that CS, when properly treated, can serve as excellent raw material for manufacturing high-quality aggregates [2,3,4,5,6]. By converting the solid waste into high value-added construction materials through innovative processing methods, this approach not only mitigates environmental burdens but also reduces the dependence on natural aggregates, aligning with the principles of sustainable development. Therefore, developing advanced techniques for fabricating solid waste-based RAs holds significant scientific and practical importance, potentially revolutionizing how the construction industry manages its waste streams while meeting growing material demands. Extensive research has been conducted on repurposing construction and demolition waste (CDW) into RAs, with methods spanning sintering [7], cold bonding [8], and alkali activation [9]. Sintered aggregates achieve high mechanical strength through vitrification at elevated temperatures, whereas cold-bonded aggregates utilize cementitious binders to circumvent energy-intensive processes. While these methods demonstrate feasibility, they face inherent trade-offs: sintered RAs contradict sustainability goals due to excessive energy demands, while non-sintered RAs often exhibit high water absorption and inadequate mechanical performance—key limitations for structural applications.

In this context, geopolymer technology [10] has emerged as a sustainable alternative, leveraging industrial by-products (e.g., fly ash, slag) to produce RAs with reduced carbon footprints. Recent studies have optimized geopolymer RAs through tailored activation strategies. For instance, Wang et al. [11] demonstrated that hybrid activators (Na_2_CO_3_/Na_2_SiO_3_) enhance reactivity and reduce costs compared to pure Na_2_SiO_3_ systems. While Du et al. [12] revealed that an optimal Ca/Al ratio promotes the co-formation of C-S-H (calcium silicate hydrate) and N-A-S-H (sodium aluminosilicate hydrate) gels, improving both aggregate stability and interfacial bonding with asphalt.

However, these advancements remain largely confined to controlled lab conditions. Critical gaps persist in scaling up geopolymer RAs derived from heterogeneous CS, where variable composition and impurities may destabilize gel formation. Moreover, the broader debate—balancing performance with sustainability—remains unresolved: Can CS be transformed into high-performance RAs without resorting to energy-intensive processes? This question underscores the need for innovative approaches that reconcile mechanical robustness, low energy input, and waste compatibility.

This study pioneers the development of structural geopolymer concrete incorporating the constructions spoil (CS)-based recycled aggregates (RAs), offering a sustainable alternative to natural aggregates. We systematically evaluate two scalable manufacturing methods-disc pelletization (producing spherical RAs) and crushing granulation (yielding angular RAs)—to assess their influence on aggregate characteristic and concrete performance. Key findings reveal that geopolymer concrete specimens incorporating spherical (disc-pelletized) CS-RAs achieve 31.0–32.5 MPa compressive strength at 7 days, while angular (crushed) CS-RAs delivers superior compressive strength (37.9–38.4 MPa), meeting structural-grade requirements. Beyond demonstrating technical viability, this work validated the environmental–economic benefits of high-value added applications for CS in structural building materials, while reducing the carbon footprint compared to conventional natural aggregates. These findings provide crucial insights for implementing circular economy principles in construction, offering a practical pathway for transforming CS from an environmental liability into a high-value resource. The study provides critical insights for implementing circular economy principles in construction, presenting a scalable pathway to transform CS from an environmental burden into a structural resource. These outcomes align with global sustainability agendas, including China’s ‘zero-waste cities’ initiative and international efforts to decarbonize the built environment.

## 2. Materials and Methods

The selection of construction spoil (CS) and ground granulated blast furnace slag (GGBS) as primary materials was based on three key considerations: (1) Sustainability—these industrial by products offer significant environmental benefits by reducing virgin material consumption and landfilling; (2) Technical synergy—GGBS provides high reactivity for alkali activation while CS serves as an effective supplementary filler with potential pozzolanic activity; and (3) Practical relevance—both materials are abundantly available in construction projects, with CS representing typical excavation waste and GGBS being widely accessible in steel-producing regions. This combination addresses both waste valorization and performance optimization objectives.

For SEM analysis, imaging was performed using a scanning electron microscope (Specification: Hitachi SU-8010, Manufacturer: Hitachi, Tokyo, Japan) operated at 5 kV accelerating voltage with a working distance of 8 mm.

XRF measurements were carried out using UniQuant Thermo Fisher Scientific spectrometer (Specification: Fluoro Max-4, Manufacturer: Thermo Fisher Scientific, Waltham, MA, USA) equipped with a 50 W Gd-anode X-ray tube. Samples were prepared as fused beads using a 1:10 sample-to-flux ratio of lithium tetraborate.

For XRD characterization, samples were analyzed using a X-ray diffractometer (Specification: SmartLab 3kW, Manufacturer: Rigaku Corporation, Tokyo, Japan) with Cu-Kα radiation (λ = 1.5418 Å) operated at 40 kV and 40 mA. Scans were collected from 5° to 80° 2θ with a step size of 0.02° and a scan speed of 2° 2θ/min.

### 2.1. Materials

**Figure 1:**
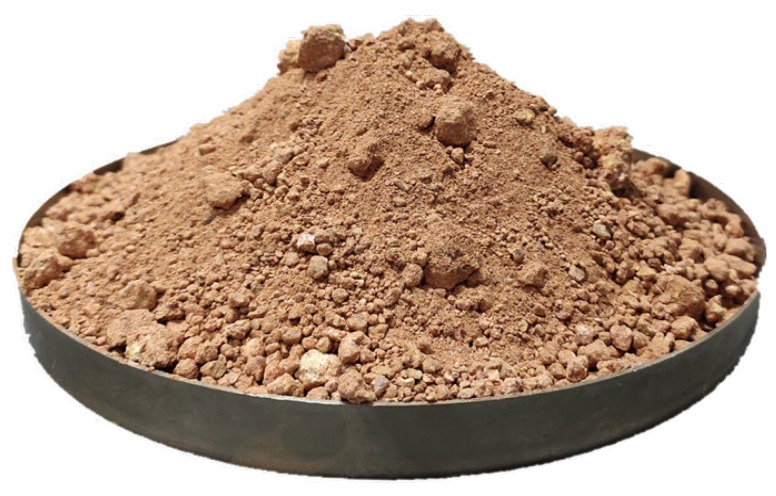
CS appearance.

**Figure 2:**
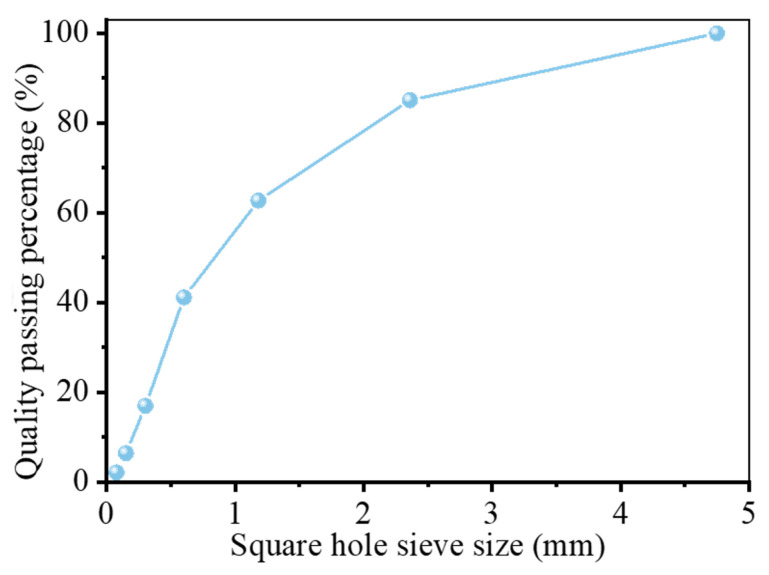
CS PSD analysis.

**Figure 3:**
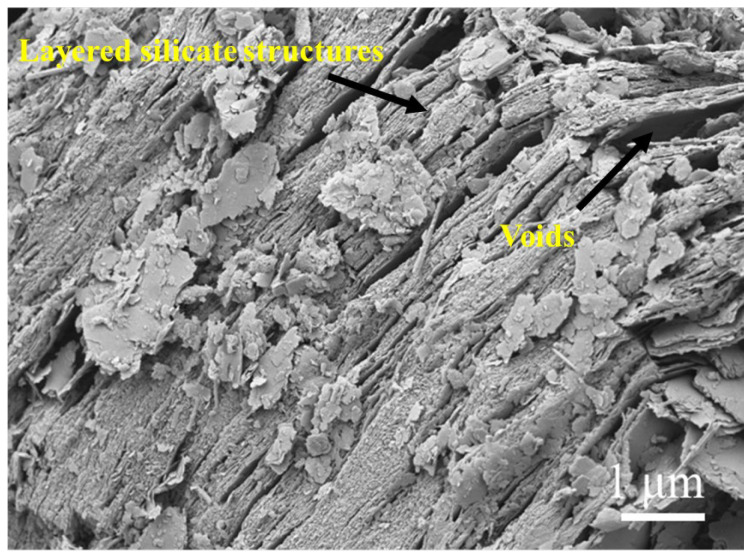
CS SEM image.

**Figure 4:**
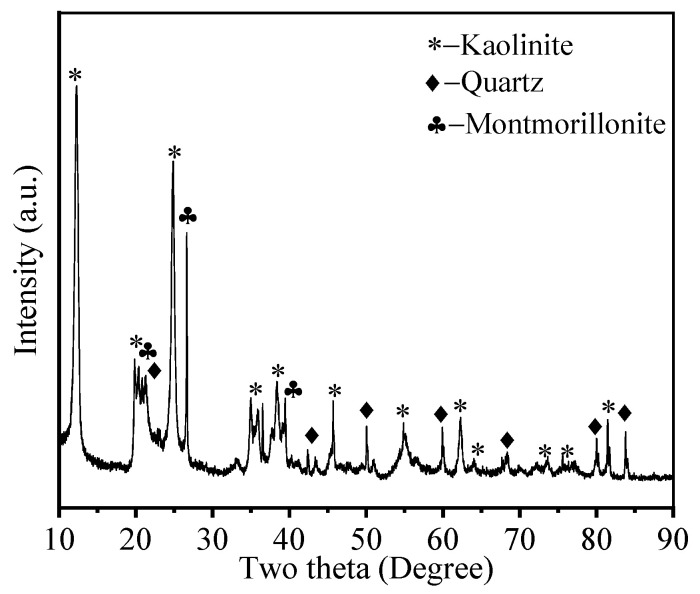
XRD image of CS [13].

The experimental materials consisted primarily of CS, GGBS, sodium silicate solution, standard sand, and deionized water. The CS was collected from the construction site of Shenzhen Longgang Metro Line 16 Phase II. As illustrated in Figure 1, Figure 2 and Figure 3, the as-received CS sample was sieved through a square mesh sieve with a normal aperture size of 1.25 mm to eliminate impurities such as small stones, and the residue after sieving was reserved for subsequent testing. SEM analysis shows that the SEM image shows typical layered silicate structures and voids inside the CS, with obvious gaps between the layers. Mass-based analysis revealed that particles below 1.25 mm constituted 79.30% of the raw sample by mass, with a moisture content of 1.47%. Through X-ray fluorescence (XRF) analysis, the chemical composition is obtained, as presented in Table 1. The CS predominantly consisted of SiO_2_ (58.05%) and Al_2_O_3_ (34.08%), collectively accounting for 92.13% of the total oxide mass. Minor constituents included Fe_2_O_3_ (5.63%), MgO, TiO_2_, and K_2_O. X-ray diffraction (XRD) analysis (Figure 4) revealed that the crystalline phase composition of the CS was dominated by kaolinite, quartz, and montmorillonite, with diffraction patterns exhibiting strong correspondence to reference standards from the International Centre for Diffraction Data (ICDD) database. These mineralogical characteristics align with the phase assemblage reported by Xiao [6] and Wang [13], confirming the prevalence of aluminosilicate phases within CS suitable for geopolymerization.

The ground granulated blast furnace slag (GGBS) used in this study, classified as grade S105 according to GB/T 18046-2017 [14] (equivalent to EN 15167-1:2008 [15]), was procured from a steel manufacturer. Quantitative X-ray fluorescence (XRF) analysis determined its chemical composition (Table 1) to consist predominantly of CaO (38.30 ± 0.45 wt%), SiO_2_ (34.20 ± 0.38 wt%), and Al_2_O_3_ (16.70 ± 0.25 wt%), with minor MgO (5.01 ± 0.12 wt%) content—a composition characteristic of high-reactivity slag suitable for alkali-activated systems. The measured CaO/SiO_2_ molar ratio of 1.12 and basicity coefficient [(CaO + MgO)/(SiO_2_ + Al_2_O_3_)] of 0.89 confirm its hydraulic potential, consistent with S105 grade specifications requiring >95% glass content and <1.5% crystalline phases.

The alkaline activator was a sodium silicate solution (Na_2_O·nSiO_2_) with a modulus (SiO_2_/Na_2_O ratio) of 1.0. Standard ISO sand (0.08–2 mm) was used as a fine aggregate reference, while deionized water ensured minimal impurity interference in the geopolymer reactions.

### 2.2. Methods

To systematically evaluate the morphology-performance relationship of recycled aggregates (RAs) in geopolymer concrete (GC), two distinct fabrication methods were employed: disc granulation (producing spherical RAs) and crushing granulation (yielding angular RAs).

#### 2.2.1. Experimental Design

Based on preliminary experiments, the mass ratio of CS:GGBS is defined as 1:1, as it ensures sufficient geopolymerization while maximizing construction spoil (CS) content, aligning with waste valorization goals. Higher GGBS proportions (>50%) accelerate setting, leading to rapid hardening that compromises workability and induces microcracking due to shrinkage stresses [16]. Conversely, excess CS (>50%) reduces strength due to its lower reactivity and layered silicate structure, which absorbs water and hinders slurry fluidity [6]. For Na_2_O concentration, a 6.5% is selected, as lower Na_2_O (<5%) inadequately activates precursors, yielding weak gels, while higher concentrations (>8%) promote efflorescence (alkali leaching) and increase carbon footprint [17,18]. A 6.5% Na_2_O equivalent optimizes activation without excessive NaOH/Na_2_SiO_3_ use, reducing costs and CO_2_ emissions from alkali production [18]. For activator modulus, 1.0 is selected, as a modulus (SiO_2_/Na_2_O molar ratio) of 1.0 balances viscosity and reactivity. High modulus (>1.2) increases viscosity, hindering spray uniformity during pelletization, while low modulus (<0.8) lacks soluble silicates for gel formation [19].

The raw material proportions for disk pelletization and crushing granulation are presented in Table 2. The mix proportion for geopolymer concrete is illustrated in Table 3. S1 and S2 refer to the spherical RAs produced by disc granulation method, C1 and C2 refer to the crushed RAs produced by crushing granulation. N indicates the natural coarse aggregate, N0 refer to the control mix without coarse aggregates.

#### 2.2.2. RAs and GC Fabrication Process

**Figure 5:**
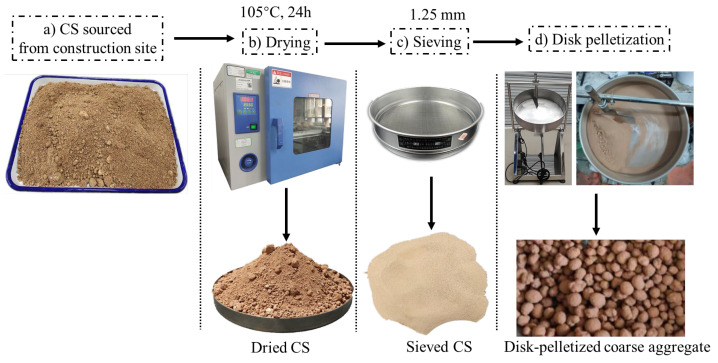
Fabrication of CS-based RAs using the disc granulation method.

For disc granulation, a mechanized pelletizer process (Figure 5) was used to fabricate spherical RAs. The process involves three stages—nucleation, layering, and spheroidization—controlled by three key parameters: rotational speed, disk inclination angle, and water spray rate. Rotational speeds above 55 rpm produce finer particles (<5 mm) due to reduced interparticle contact time and increased collision frequency, while lower speeds (30–40 rpm) enable layer-by-layer growth, forming larger particles (8–15 mm). The disk inclination angle was optimized at 45°, as angles below 40° reduce sphericity and angles above 55° cause material buildup at the disk base, disrupting pellet formation. The water spray rate was carefully controlled to a water-to-solid ratio of 0.285, avoiding overwetting (which forms oversized agglomerates or slurry) or insufficient spray (which results in weak granules).

For crushing granulation, prismatic mortar specimens (40 mm × 40 mm × 160 mm) were first prepared according to the mix proportions in Table 2, and sealed-cured for 7 days (20 ± 2 °C, RH ≥ 95%) to promote geopolymerization. The specimens were then mechanically crushed using a jaw crusher (Specification: PE-150×250; Manufacturer: ZhuoHui Machine, Shenzhen, China, discharge setting: 5–20 mm), yielding angular aggregates with higher surface texture complexity compared to the spherical pellets from disc granulation. This morphology is expected to enhance mechanical interlocking at the aggregate-cement paste interface, improving concrete’s compressive strength and crack resistance. The crushed material was sieved into 5 mm, 10 mm, and 20 mm fractions to obtain the 5–20 mm grade, then air-dried (25 °C) to ≤2.0% moisture before storage. This gradation complies with GB/T 14685-2022 standards [20] for coarse aggregates, ensuring proper particle size distribution (PSD), flakiness limits, and crushing resistance.

For geopolymer concrete fabrication, the cubic specimens (100 mm × 100 mm × 100 mm) were prepared using the two types of RAs, as well as natural aggregates for comparison. The mix proportions are detailed in Table 3. During geopolymer concrete fabrication, the solid precursor materials (CS, GGBS) were first dry-mixed in a horizontal mixer for two minutes to ensure homogeneity. Subsequently, the alkaline activator solution was gradually added and mixed for an additional three minutes to initiate geopolymerization. Finally, the remaining mixing water was introduced, and blending continued until a uniform fresh mixture was achieved. After casting, the specimens were compacted on a vibrating table to eliminate entrapped air bubbles, ensuring optimal mechanical performance.

#### 2.2.3. Testing Methods

The particle size distribution (PSD) of recycled aggregates (RAs) was determined via sieve analysis in accordance with ASTM C136/C136M [21]. A representative sample of RAs (approximately 2 kg) was air-dried to a constant moisture content (≤2.0%) and sieved using standard sieves with aperture sizes of 5 mm, 10 mm, and 20 mm. The sieving process was performed using a mechanical shaker for 10 min, and the mass retained on each sieve was recorded to calculate the cumulative percentage passing. The PSD curve was checked for compliance with the requirements of GB/T 14685-2022 for coarse aggregates.

The workability of fresh geopolymer concrete (GC) was evaluated using the slump test, conducted in accordance with ASTM C143/C143M [22]. A standard slump cone (height: 300 mm, top diameter: 100 mm, bottom diameter: 200 mm) was filled in three equal layers, with each layer rodded 25 times using a 16 mm diameter tamping rod. After removing the cone vertically, the difference in height between the mold and the displaced concrete was measured to the nearest 5 mm. The slump was controlled to a target value of 200 ± 10 mm, ensuring optimal workability for general concrete applications.

The compressive strength of GC was tested strictly in accordance with GB/T 50081-2019 [23]. Cubic specimens (100 mm × 100 mm × 100 mm) were cast, compacted on a vibrating table, and sealed-cured at 20 ± 2 °C and relative humidity (RH) ≥ 95% for desired duration. The specimens were then tested using a hydraulic compression testing machine (Specification: EH-5305; Manufacturer: Shenzhen Enpuda Industrial System Co., Ltd., Shenzhen, China) with a capacity of at least 2000 kN, applying a constant loading rate of 0.5 MPa/s until failure. The compressive strength (*f*_c_) was calculated as the ratio of the maximum load (*F*) to the cross-sectional area (*A*), ensuring accurate and reliable results.

## 3. Results and Discussion

### 3.1. RAs Characterization

**Figure 6:**
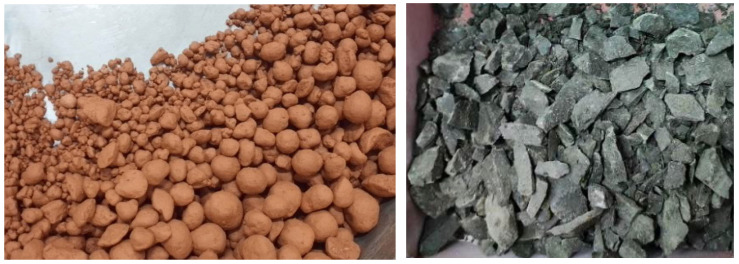
Appearance of the disc-pelletized and crushed CS-based RAs.

The recycled aggregates (RAs) exhibited distinct morphological and particle size distribution (PSD) characteristics depending on their production method. As shown in Figure 6, disc-pelletized RAs (S1, S2) displayed spherical geometries with smooth surfaces due to rolling and compaction forces during granulation, while crushed RAs (C1, C2) had irregular, angular shapes resulting from mechanical fracturing. The pelletized RAs appeared lighter in color, indicating partial vitrification, whereas crushed RAs retained a darker, heterogeneous hue, reflecting their unprocessed raw material composition. These morphological differences are expected to influence workability, interfacial bonding, and mechanical performance in geopolymer concrete.

**Figure 7:**
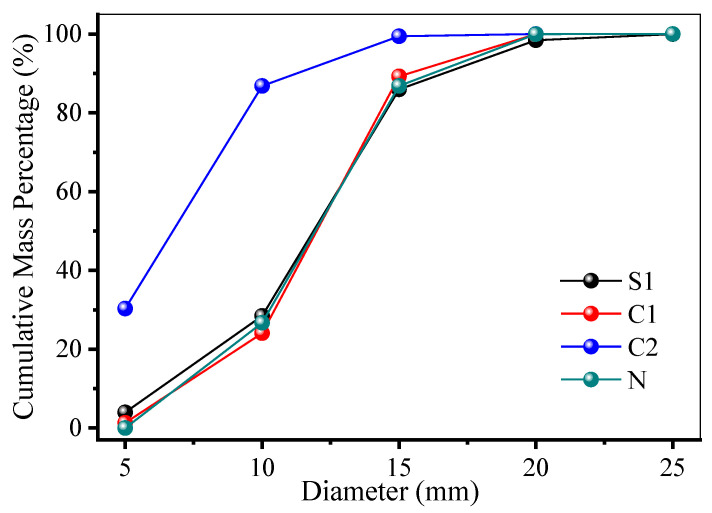
Particle size distribution comparison of the RAs and natural aggregates.

The particle size distribution (PSD) analysis (Figure 7) further highlighted key variations. Pelletized RAs (S1) exhibited a steep PSD curve, with 28.45% passing a 10 mm sieve and 86.81% passing a 15 mm sieve, indicating a narrow, uniform size range that enhances packing density and workability. In contrast, crushed RAs (C2) showed a broader distribution, with 30.30% passing a 5 mm sieve, 86.72% passing a 10 mm sieve and 99.45% passing a 15 mm sieve, suggesting higher internal friction and potential workability challenges. Natural aggregates (N) demonstrated an intermediate, well-graded distribution, with 26.70% passing a 10 mm sieve and nearly 100% passing a 20 mm sieve. The angularity and rough texture of crushed RAs may improve mechanical interlocking and compressive strength, while the spherical shape of pelletized RAs could enhance flowability. These findings emphasize the importance of selecting aggregates based on desired concrete properties, with pelletized types favoring workability and crushed types offering potential strength benefits.

### 3.2. GC Characterization

**Figure 8:**
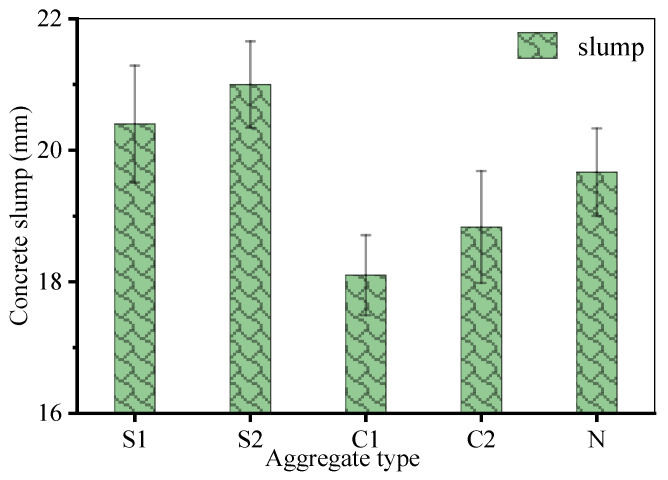
Geopolymer concrete slump measurement results.

The workability of geopolymer concrete, evaluated through concrete slump tests, varied significantly with aggregate type (Figure 8). Mixes incorporating pelletized RAs (S1/S2) achieved the target slump range of 200 ± 10 mm with a water-to-binder ratio of 0.35, owing to the spherical shape and smooth surface texture of the RAs, which reduced internal friction. In contrast, mixes with crushed RAs (C1/C2) required 5–7% more water (w/b = 0.37) to attain comparable workability, a consequence of angular particle morphology and surface roughness increasing mix resistance. The natural aggregate (N) exhibited intermediate behavior, requiring w/b = 0.36 to meet the target slump, reflecting its well-graded but less optimized particle shape.

Failure analysis revealed distinct fracture mechanisms: (1) Pelletized RAs (S1/S2): Specimens predominantly failed through cohesive cracking within geopolymer paste, indicating strong interfacial bonding between the smooth aggregate surfaces and the matrix. (2) Crushed RAs (C1/C2): Failure occurred via interfacial debonding, likely due to stress concentrations around angular edges and microcracks induced by surface roughness.

These results underscore the trade-off between workability and interfacial strength. While pelletized RAs enhance flowability with lower water demand, crushed RAs may improve mechanical interlocking—albeit at the cost of higher water requirements and potential durability concerns from microcracking.

**Figure 9:**
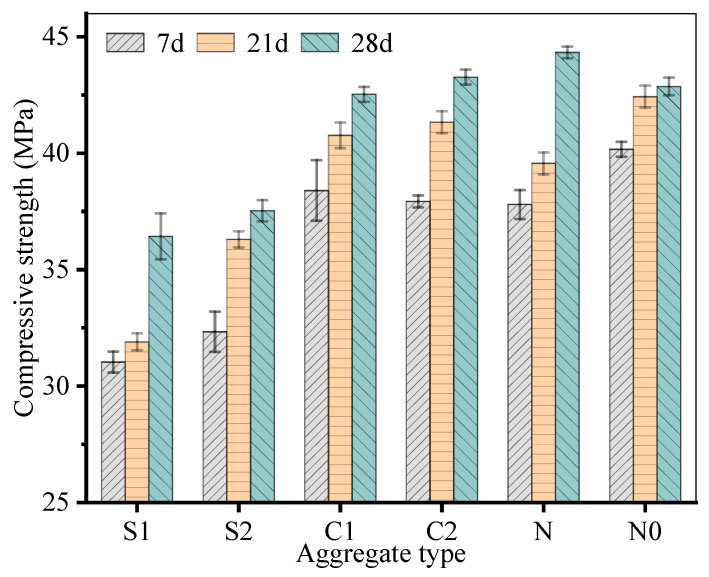
Compressive strength development of geopolymer concrete with different aggregates.

The compressive strength of geopolymer concrete, as illustrated in Figure 9, was evaluated at 7, 21, and 28 days for various aggregate types: S1 and S2 (disc-pelletized RAs), C1 and C2 (crushed RAs), N (natural aggregate), and N0 (control mix without aggregates).

As shown in Figure 9, at all testing ages, concrete specimens with disc-pelletized RAs (S1 and S2) demonstrated lower compressive strengths compared to that with other aggregates. For instance, at 28 days, concrete with disc-pelletized RAs S1 and S2 achieved strengths of 36.9 MPa and 37.1 MPa, respectively. In contrast, concrete with crushed RAs C1 and C2 achieved strengths of 42.3 MPa and 43.5 MPa. This suggests that the spherical morphology and smooth surface of the pelletized RAs may not provide optimal interfacial bonding with the geopolymer matrix. This phenomenon has also been reported by references [24,25].

The angular shape and rough texture of these aggregates likely enhance mechanical interlocking and improve the bond between the aggregate and the surrounding matrix, confirmed by reference [26].

The natural aggregate (N) showed a balanced performance, achieving a 44.3 MPa compressive strength at 28 days. This result aligns with the well-graded particle size distribution and favorable microstructural properties of natural aggregates, which contribute to effective load transfer within the concrete. Notably, the control mix (N0) without aggregates displayed the 42.7 MPa compressive strength at 28 days. This observation underscores the significant role of aggregate type and morphology in influencing the mechanical properties of geopolymer concrete. The absence of aggregates allows for a more homogenous matrix structure, potentially leading to superior strength.

Overall, the choice of aggregate type significantly impacts the compressive strength of geopolymer concrete. While disk-pelletized aggregates offer advantages in terms of workability and uniformity, they may compromise strength compared to crushed or natural aggregates. Further research is warranted to optimize the balance between aggregate characteristics and concrete performance. The results suggest that with further optimization of pelletization parameters (e.g., compaction pressure, binder content), recycled aggregates could potentially match or exceed natural aggregate performance while offering sustainability benefits. The findings also highlight the importance of considering both aggregate morphology and size distribution when designing geopolymer concrete mixes, as these factors collectively influence fresh properties, hardened characteristics, and microstructural development.

## 4. Conclusions

This study systematically evaluated the performance of geopolymer concrete using different types of aggregates, including disc-pelletized (S1 and S2), crushed (C1 and C2), natural (N), and a control mix without aggregates (N0). The findings provide valuable insights into the influence of aggregate morphology and particle size distribution on the mechanical properties and workability of geopolymer concrete.

(1) The disc-pelletized RAs (S1 and S2) exhibited a well-rounded, spherical shape with a narrow particle size distribution, resulting in improved workability but lower compressive strength compared to other aggregate types. This can be attributed to their smooth surface and reduced interfacial bonding with the geopolymer matrix. In contrast, the crushed RAs (C1 and C2) demonstrated higher compressive strengths due to their angular shape and rough texture, which enhance mechanical interlocking and bond formation. The natural aggregate (N) showed a balanced performance, achieving comparable strength to the crushed RAs while maintaining a well-graded particle size distribution.

(2) At 28 days, the control mix (N0) achieved the highest compressive strength (44.3 MPa), highlighting the significant role of aggregate type in determining concrete performance. The crushed RAs (C1 and C2) followed closely with compressive strengths of 42.3 MPa and 43.5 MPa, respectively. The natural aggregate (N) also performed well, reaching 42.7 MPa. However, the disk-pelletized RAs (S1 and S2) exhibited lower strengths (36.9 MPa and 37.1 MPa), indicating that their morphology may not be optimal for maximizing strength in geopolymer concrete.

In conclusion, this study provides a comprehensive understanding of the impact of aggregate type on geopolymer concrete properties, contributing to the development of sustainable construction materials with tailored performance characteristics.

## Figures and Tables

**Table 1:** XRF analysis of the CS and GGBS (wt%).

Materials	SiO_2_	Al_2_O_3_	CaO	Fe_2_O_3_	MgO	K_2_O	TiO_2_	Na_2_O	SO_3_	P_2_O_5_	LOI
CS	58.05	34.08	0.03	5.63	0.42	0.66	0.77	0.04	0.11	0.04	0.17
GGBS	34.20	16.70	38.30	1.50	5.01	0.40	0.83	0.58	1.24	-	1.24

**Table 2:** Mix proportions for RAs production through disc granulation and crushing granulation.

Label	GGBS (g)	CS (g)	Na_2_SiO_3_ (g)	H_2_O (g)	Sand (g)	Curing Age (d)
S1	500	500	127.9	285	-	1
S2	500	500	127.9	285	-	7
C1	500	500	127.9	450	0	7
C2	500	500	127.9	450	1000	7

**Table 3:** Mix proportions for geopolymer concrete.

Type	GGBS (g)	CS (g)	Na_2_SiO_3_ (g)	H_2_O (g)	Sand (g)	Aggregate Mass (g)
S1	2700	2700	690.68	2700	5400	8100
S2	2700	2700	690.68	2700	5400	8100
C1	2700	2700	690.68	2700	5400	8100
C2	2700	2700	690.68	2700	5400	8100
N	2700	2700	690.68	2700	5400	8100
N0	4050	4050	1036.02	4050	8100	0

## Data Availability

The original contributions presented in this study are included in the article. Further inquiries can be directed to the corresponding author.

## Abbreviations

The following abbreviations are used in this manuscript:

CDW	Construction and demolition waste
CS	Construction spoil
GGBS	Ground granulated blast furnace slag
PSD	Particle size distribution
GC	Geopolymer concrete
RA	Recycled aggregate
